# Secondary School Male Students Perception Towards Their Weight, Almethnab Town, Qassim, Saudi Arabia

**DOI:** 10.7759/cureus.20370

**Published:** 2021-12-12

**Authors:** Omer A AsSaigal, Suliman Alguefly, Yousif M El-Mosaad, Abdullah M Al Saigul

**Affiliations:** 1 Diabetes and Endocrinology, King Saud Hospital, Unaizah, SAU; 2 Public Health, Almethnab Health District, Almethnab General Hospital, Almethnab, SAU; 3 College of Applied Medical Sciences, King Faisal University, Al Ahsa, SAU; 4 Family Medicine Academy, Qassim Health Cluster, Buraidah, SAU

**Keywords:** obesity, adolescents, health promotion, lifestyle, weight perception

## Abstract

Objective

To determine the perception of Almethnab town secondary school male students towards their body weights.

Methodology

We surveyed male secondary school students in Almethnab town in Qassim using a self-administered questionnaire and weighing/height scale. All of the four secondary schools were targeted. The calculated sample size was 256 students. A stratified cluster sampling technique was used to select research participants. Body mass index (BMI) was calculated and interpreted using web-based CDC childhood and adolescent BMI calculator. Weight status perception was assessed by comparing students' opinions about their weight against BMI percentile status.

Results

Out of 363 selected students, all of the available 270 students participated, with a response rate of 74%. Thirty-two (12.2%) of the students were underweight, 143 (54.6%) were normal, 30 (11.5%) were overweight, and 57 (21.8%) were obese. Students perception towards their weight was correct for 159 (65%) of students. Out of the 87 overweight/obese students, 16 (18.4%) perceived their weight as appropriate, 17 (53.1%) of 32 underweight students look at their bodies as normal, while 29 (20.4%) of normal weight students perceive themselves as overweight. Obese students exercise less both in frequency and duration than non-obese ones. Sweetened drinks, fast food and frequent snacks are weakly associated with obesity.

Conclusion

Prevalence of obesity is very high among male secondary school students in Almethnab town.One-third of students have misperceptions about weight status.

## Introduction

Obesity is a major risk factor for chronic diseases and a leading cause of premature death. Metabolic diseases, cardiovascular diseases, some cancers, and musculoskeletal disorders association with obesity are well documented. Global obesity prevalence has been doubled since 1980. Obesity epidemicity makes it one of the most serious modifiable health and economic challenges of the early 21st century [1].

Childhood obesity and raised body mass index (BMI) are associated with a higher chance of adult obesity and chronic diseases [1]. Increasing rates of overweight and obesity among children and adolescents are of particular regional and international concern [1-5].

Obesity is a complex behavioral problem that has individual, family, societal, and environmental dimensions [2]. Excess calorie intake and sedentary lifestyle are the main two factors behind obesity in most cases [6].

Prevalence of obesity among adolescents is less investigated compared to children and adults. Obesity prevalence had reached epidemic levels in the kingdom with a quarter of adult males and a third of females are overweight or obese [7]. Among Saudi children and adolescents, previous studies had shown high prevalence of overweight and obesity in the kingdom. About a quarter to one-third of Saudi adolescents were found overweight or obese [8,9]. Furthermore, around half of Saudi male adolescents were found to be physically inactive [10] and consume a lot of sugary drinks [10,11].

Saudi Arabia is a developing country with a young population as about one of five nationals is an adolescent. Health promotion and disease prevention is a lifetime activity. The early recognition of chronic diseases risk factors is the basic element of preventive plans. Similarly, determining population attitude towards the health issue of interest is a pre-requisite for proper intervention activities. 

Misperception of Weight was found to be common among children and adolescents [8,11-13]. Behavioral change starts with s positive attitude. Appropriate weight control behaviors in youth had been linked to accurate weight status self-perception.

The objectives of this study were to estimate the prevalence of obesity among male secondary school students in Almethnab town, to determine students perception towards their weight, and to describe students exercise and dietary habits related to weight status.

## Materials and methods

Almethnab town is located in Qassim province with about 30,000 inhabitants. We surveyed all of the four male secondary schools at Almethnab town. Of 781 male students, a stratified cluster sampling technique was used to select research participants. The sample size was calculated using the prevalence rate of overweight and obesity among Saudi adolescents from previous studies (around 30%) [9], confidence limit 5%, and design effect of 1.0%.

The calculated sample was 265. All of the four schools participated in the study. One to two classes per level were randomly chosen from each school according to the school size. At each class, all available students were requested to participate in the study. A self-administered semi-structured questionnaire was completed by every participant, then weight and height were recorded by the investigators using a daily calibrated standardized scale.

The questionnaire included demographic data, family history of obesity, dietary, and exercise habits. Each student was asked to write down his weight and height, if he knew them and to choose his perception about his weight status from a list of four items. The items were appropriate, underweight, overweight, and obese. Dietary questions included questions about eating habits, e.g. number of daily meals, snacks, and weekly fast food intake, sweetened drinks daily and weekly. Students were also asked if they exercise regularly and to report the duration and frequency for each exercise type. Four common types of locally favored sports were included; football, jogging, gym sports, and walking. Other sports were grouped as others. Sleep duration and time spent on TV and mobile phone were also recorded. 

BMI was calculated and interpreted using web-based CDC childhood and adolescent BMI calculator. BMI percentile for age was categorized to the following: underweight (<5th percentile), normal weight (5th -85th percentiles), overweight (86th -95th percentiles), and obese (>95th percentile) [14].

We could not use the national BMI for age graph developed by AlHerbish [15] to assess students' weight status as it has to be used manually for each case by plotting BMI reading on the graph. The CDC interpretation tool is expected to overestimate the obesity prevalence [8]. Data were entered and analyzed using Epi Info 3.5 software program.

Weight status perception was assessed by comparing students' opinion about their weight against BMI percentile status. Overweight and obesity are combined to become one group for perception and frequency of associating factors. At each class, the attending investigators presented themselves, explained the questionnaire, and invited the students to participate. Furthermore, the questionnaire included an information sheet that described the purpose of the questionnaire and emphasized the voluntariness of participation. Once the questionnaire is completed by each student, individual result was conveyed privately by one of the investigators and the necessary advises were given. The study was approved by the regional research ethics committee, approval number 20170506. The committee is registered at the national bioethics committee, registration number H-04-Q-001. Permission to implement it was granted by the provincial education directorate. 

## Results

Out of all of the four male secondary schools in Al Methnab town, we targeted 15 classes. In these classes, there were 363 registered students. All of the 270 available students participated in this survey. This gave a response rate of 74%. All students completed the questionnaire, but four of them left before measuring their weight and height.

Table 1 summarizes the main sample characteristics, weight status and weight perception. Age range is 6 (15-21) years, but only 18 students were more than 18 years. Most of the students (88.1%) were Saudi nationals. Thirty students (11.1%) reported having asthma while only 2 are diabetics and one is hypertensive. Six students have other chronic diseases.

**Table 1:** Participant characteristics, weight perception and lifestyle variables by weight percentiles, Al-Methnab Male Secondary Schools.

Item	Underweight		Normal		Overweight/Obese		Total
	No	(%)	No	(%)	No	(%)	No
Weight Perception							
Appropriate	17	14.7	83	71.6	16	13.8	116
Underweight	10	31.3	22	68.8	0	0.0	32
Overwt Obese	0	0.0	29	30.5	66	69.5	95
Don’t Know	5	27.8	8	44	5	27.8	18
TOTAL	32	12.2	142	54	87	33.3	261
Age							
15	5	21.7	8	34.8	10	43.5	23
16-17	14	10.0	81	57.9	45	32.1	140
>=18	6	9.7	33	53.2	23	37.1	62
TOTAL	25	11.1	122	54.2	78	34.7	225
Nationality							
Non-Saudi	0	0.0	12	70.6	5	29.4	17
Saudi	30	12.6	128	53.8	80	33.6	238
Unknown	2	28.6	3	42.9	2	28.6	7
TOTAL	32	12.2	143	54.6	87	33.2	262
Family Hx of Obesity							
Yes	10	8.1	74	59.7	40	32.3	124
No	22	16.2	69	50.7	45	33.1	136
TOTAL	32	12.3	143	55.0	85	32.7	260
Daily Snacks							
<2	14	14.7	52	7	29	30.5	95
>=2	16	10.7	80	53.7	53	35.6	149
TOTAL	30	12.3	132	54.1	82	33.6	244
Daily Fast food							
<2	11	13.8	48	60.0	21	26.3	80
>=2	20	11.4	91	51.7	65	36.9	176
TOTAL	31	12.1	139	54.3	86	33.6	256
Sweetened Drinks/Week							
<3	11	19.0	32	55.2	15	25.9	58
>=3	8	9.5	40	47.6	36	42.9	84
TOTAL	19	13.4	72	50.7	51	35.9	142
Exercise							
Yes	12	10.9	67	60.9	31	28.2	110
No	20	13.2	76	50.0	56	36.8	152
TOTAL	32	12.2	143	54.6	87	33.2	262
Weekly Sport (Hours)							
0	16	13.2	63	52.1	42	34.7	121
1—2	4	12.9	17	54.8	10	32.3	31
>2	6	12.2	29	59.2	14	28.6	49
TOTAL	26	12.9	109	54.2	66	32.8	201
Daily Sleep Duration							
(Hours)							
<6	7	19.4	15	41.7	14	38.9	36
6—8	16	10.1	94	59.1	49	30.8	159
>=9	9	13.6	33	50.0	24	36.4	66
TOTAL	32	12.3	142	54.4	87	33.3	261
Daily TV Mobile phone use							
(Hours)							
<4	13	18.6	35	50.0	22	31.4	70
>=4	19	10.2	103	55.4	64	34.4	186
TOTAL	32	12.5	138	53.9	86	33.6	256

About half of the students, 141 (52.2%), thought they know their weights while only 98 (36.3%) thought they know their heights. Of those who reported their weight, only 11% had the exact weight, but 55.5% were within ±3 kg from the measured weight, while only 12 (8.8%) students were within ±3 cm of their measured heights.

**Figure 1:**
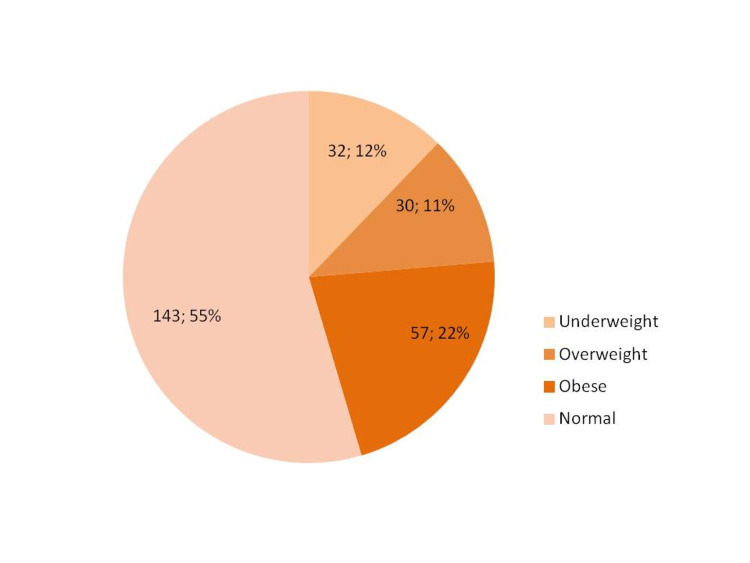
BMI percentile status for male secondary school students, Al Methnab town, Qassim, Saudi Arabia (n = 262 students).

Using CDC BMI percentile interpretation for children and teenagers, 32 (12.2%) of the students were underweight, 143 (54.6%) were normal, 30 (11.5%) were overweight, and 57 (21.8%) were obese. Overweight and obesity together comprised 87 (33.2%) students (Figure 1).

**Figure 2:**
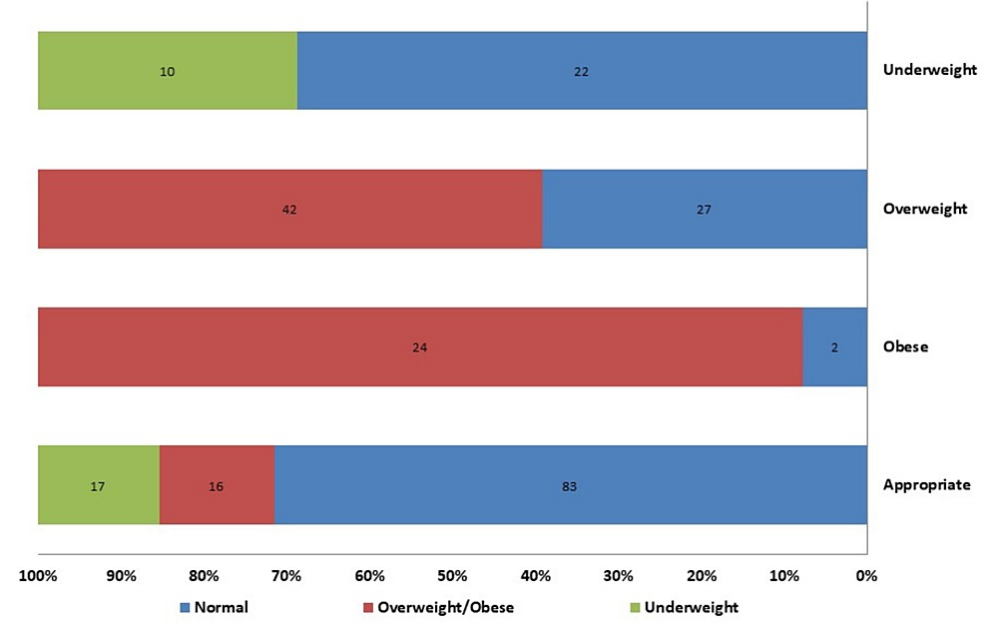
Body image perception versus BMI percentile status for male secondary school students, Al Methnab town, Qassim, Saudi Arabia (don't know group (18 students) are excluded, n = 243 students).

Students' perception towards their weight was correct for 159 (60.9%) of students. Eighteen students (6.9%) reported having no idea about their weight status. Out of the 87 overweight/obese students, 16 (18.4%) perceived their weight as appropriate, 17 (53.1%) of 32 underweight students look at their bodies as normal, while 29 (20.9%) of normal weight students perceive themselves as overweight (Figure 2).

About half of the students had no family history of obesity, 136 (51.9%). Obese students were similar to non-obese in the presence of family history of obesity, 40 (47.1%) and 86 (48.6%) respectively, OR 0.94 (P=0.8).

Of all study sample, only 112 (42.0%) reported having regular exercise. Football was practiced by 78 (28.9%) students followed by walking 28 (10.3%) then jogging and gym sports 14 (5.2%) each. For those who reported exercising regularly, the mean duration of exercise hours per week were 4.7 and 6.8 hours respectively, the difference between obese and non-obese students was not statistically significant.

Overweight/obese students were less active than normal ones; 31 (35.6%) and 67 (46.9%) respectively. The difference between obese and non-obese was not statistically significant, OR 0.6 (P-value = 0.07).

No student reported taking more than three meals per day. Six students reported having only one main meal per day, but all of them were of normal weight. Obese students were more likely to have three meals per day than non-obese ones, 65.1% and 55.6 respectively, P-value=0.07. Obese and non-obese were similar in mean fast food consumption per week; 2.6 and 2.7, respectively. However, the proportion of obese students who took two or more fast food daily was more than that of non-obese ones, 75.6% obese and 66. 5% non-obese, but the difference is not statistically significant, (OR; 1.6, CI 0.9-2.8) P=0.1. Similarly, mean snacking between main meals was 2.2 and 2.0, OR of having two or more snacks per day is 1.2 (C.I 0.8 -1.4) P = 0.5.

Of all students, 192 (76.7%) drank 3 or more sweetened drinks weekly, 73 (29.3%) reported 8 or more drinks per week. Obese and non-obese were similar in mean of drinks per week, 6.0 and 5.9, respectively. They were also similar in sleep duration (mean 7.5 and 7.3 hours.) and watching TV/mobile phone, 5.7 and 5.4 hours per day, respectively.

## Discussion

Obesity prevalence among Saudi adolescents is at an epidemic level. The prevalence of overweight/obesity among Al-Methnab male secondary students is similar to the reported levels in the Kingdom [8]. Adolescent obesity prevalence in developed countries is creating a lot of concern, yet its values are much lower than ours [1,4].

Physical inactivity in our sample is very high. If half of this generation are physically inactive today, then the future of physical activity is gloomy. Physical inactivity is more frequent among obese individuals. Although the difference between obese and non-obese had not reached statistical significance, those differences are sound. Exercise duration has a difference of 2.1 hours per week in favor of non-obese, an increase of 44.7% of weekly hours. Low physical activity had been documented among Saudi youth, with a similar prevalence of inactivity. Again local figures are worse than those of developed countries [10].

Bad dietary habits are the second main factor for obesity. Frequent reports had found high-calorie intake, high fast food and snacking among children and adolescents [10,11]. Modernization and affluence made unhealthy eating a social advantage. Availability, affordability and high media influence supported the fast transition from low consumption of natural food, to the state of overeating unhealthy high-calorie food. A similar high frequency of fast food consumption was also reported by Al Hazzaa [10], and El Moghny [11], 2.9 and 2.2 fast food weekly, respectively. The difference in frequency of fast food consumption between obese and non-obese was negligible and there were no appreciable differences between them in number of sweetened drinks consumption, sleep duration or TV/mobile phone hours spent per day. The absence of differences may be due to recall-bias, under-estimation or over-estimation of values that are not uniformly equal among the two groups.

It is important to note that more than half of our sample consume three or more sweetened drinks per week. This unhealthy practice was also found to be high by El Moghny [11], where about half of the study sample consumed seven or more cans per week and also by Al-Hazzaa [10], where two-thirds of male adolescents consume three or more sweetened drinks per week.

Long durations of TV and mobile phone use for entertainment activities had been sharply increased with the wide availability of smartphones. The negative effects on the body and mental health don't need to be overemphasized. The association between obesity and sedentary life is consolidated [1].

More than two-thirds of our sample spend four hours or more on TV/mobile phone, more than double the recommended time by The American Academy of Pediatrics and also much more than Al-Hazzaa study [10]. The reason is possible that those studies were conducted before the mobile phone technology advancement and abundance.

Adolescent knowledge about their weight and perception towards their weight are important. About half of our sample did not know their weights and two-thirds did not know their heights. Compared to adults, misperception of body image among adolescents was not extensively studied. Misperception about own weight was seen in one-third of our sample. Of normal BMI for age, 22 (15.8%) felt that they are underweight while 29 (20.8%) over-estimated their weight status. Eleven (36.7%) of overweight ones thought that their weight is appropriate. Furthermore, few other obese boys, 5 (8.8%) looked at their weights as appropriate. This is serious as the true perception of own image is mandatory for behavioral change. An American study found high misperception of weight among children 8-15 years. Majority of overweight and about half of obese boys feels that their weight is "about right" [12]. The difference in age spectrum may explain the large differences between the two studies.

Our sample had worse weight perception compared with Riyadh male adolescents study by Al Rukban [8], but better than the Dubai study by Musaiger [13]. Al Rukban study was conducted more than 10 years ago, a point reflecting unimproved weight perception of the new generation.

Parents attitude is of paramount importance in obesity. Underestimation of body weight among children's parents was reported locally [16] and internationally [17]. Children's parents in Qassim misclassified most of overweight and more than half of obese children [16]. Although we have not studied parents' attitudes, any intervention targeting obesity must involve adolescent parents.

World health organization and The Regional Eastern Mediterranean Office (EMRO) are facing the escalating prevalence rates of adolescent obesity with multidimensional approaches and strategies [2,5,6]. Unless these strategies are seriously adopted by the concerned governmental and non-governmental organizations, the future of this generation is worrisome.

Due to the limitation of staff and time constraints, the surveyed population was limited to male adolescents above 14 years of age who are enrolled in Almethnab secondary schools. As we couldn't survey female students, we were unable to generalize our results to female adolescents, though previous studies showed a higher prevalence of obesity among Saudi adolescents compared to males [8]. We also had used the CDC BMI calculator and interpretation tool which is expected to overestimate the obesity prevalence in our community. 

## Conclusions

Prevalence of obesity is very high among male secondary school students in Almethnab town. One-third of students had misperceptions about their weight status. Half of all of the students did not exercise regularly. Obese students exercised less both in frequency and duration than non-obese ones. Sweetened drinks, fast food and frequent snacks were weakly associated with obesity. Although our prevalence is similar to previous studies, our study was confined to male secondary school students in Al Methnab town, hence it can't be generalized outside this narrow age range, to female teenagers, nor to other communities. 

We recommend monitoring obesity prevalence by periodic surveys and screening students at schools and at primary care centers for obesity. Students' awareness about obesity and appropriate body image has to be addressed at media and schools. These programs should target students and their parents. Intensive interventional programs that target obesity risk factors, healthy eating, and regular physical activity are recommended.
